# Machine learning -based decision support framework for CBRN protection^☆^

**DOI:** 10.1016/j.heliyon.2024.e25946

**Published:** 2024-02-09

**Authors:** Tamás Kegyes, Zoltán Süle, János Abonyi

**Affiliations:** a HUN-REN-PE Complex Systems Monitoring Research Group, Egyetem utca 10., Veszprém, 8200, Hungary; b University of Pannonia, Egyetem utca 10., Veszprém, 8200, Hungary

**Keywords:** CBRN protection framework, Machine learning, Predictive analytics, Information management, Decision support system

## Abstract

Detecting chemical, biological, radiological and nuclear (CBRN) incidents is a high priority task and has been a topic of intensive research for decades. Ongoing technological, data processing, and automation developments are opening up new potentials in CBRN protection, which has become a complex, interdisciplinary field of science. According to it, chemists, physicists, meteorologists, military experts, programmers, and data scientists are all involved in the research. The key to effectively enhancing CBRN defence capabilities is continuous and targeted development along a well-structured concept. Our study highlights the importance of predictive analytics by providing an overview of the main components of modern CBRN defence technologies, including a summary of the conceptual requirements for CBRN reconnaissance and decision support steps, and by presenting the role and recent opportunities of information management in these processes.

## 1 Introduction

Chemical, biological, radiological, and nuclear (CBRN) defence activities were initially driven by the military potential of the major powers and their ongoing threats, albeit of varying intensity. Today, terrorist groups beyond the control of states are more dangerous, but our industrialised, politically, economically, and culturally conflicting ages also increase the risk of CBRN disasters. The continued development of CBRN protection capabilities is the top priority of global security [1]. Although most countries have a few decades of CBRN defence history, new directions for development are emerging in our rapidly changing world [2].

Machine learning (ML) and artificial intelligence (AI) techniques enable more complex and highly automated system developments. But there is no generally approved framework for it. Furthermore, there is no detailed overview to describe which ML methods fit the different CBRN tasks or a guideline on how to select an adequate ML method for automating CBRN steps.

This work aims to systematise the functional components of CBRN protection by reviewing the relevant literature, identifying their potential for efficiency improvement, and collecting the best machine intelligence practises that support it. We aim to prove that a modern CBRN solution definitely requires advanced machine intelligence solutions. But to achieve effective protection, long-term plans and an adequate framework are needed to support systematic developments.

The key contributions of our work are the followings:

• We gave an overview of structuring CBRN protection tasks for organizing them into a protection framework.
• We summarized the phases of ML development processes and collected advanced applications for demonstrating the potential of exploiting machine intelligence capabilities.
• We have proven that ML techniques are already widely used in several elementary CBRN protection tasks, and moreover, a modern CBRN protection system is infeasible without ML-based solutions.
• We presented a new modular framework instead of the static scenario-based CBRN protection approach. The novelty primarily comes from the central role of ML-based information management and network decision support functionalities.
• We have shown that OODA (observe-orient-decide-act) approach can be integrated both on lower-level and higher-level CBRN protection tasks, and it can support risk-based approach as well.
• Finally, we highlighted the potential to increase the overall efficiency of CBRN protection mechanism by introducing a decision-support network-based solution.

In Section 2 of this article, we describe the structure of CBRN defence, including an overview of the tools of CBRN detection and their current potential compared to the “old-style” procedure. We then describe the role and steps of information management in the processing of raw data and define some key objective functions that allow the use of optimisation techniques, which are widely used in industry, in the field of CBRN detection. In Section 2.3, we review the potential applications of machine learning in CBRN protection processes, describe the requirements of the model for data and its processing, and illustrate the efficiency-enhancing capabilities of machine learning through best practises. Then in Section 3, we take a look at Boyd's OODA loop, which suggests a methodology to follow the observe-orient-decide-act cycles. Although OODA as a general approach appears in several fields of military and defence solutions, especially in CBRN as well, there are further problems where statistical analysis can help improve the processes from threat flow modelling due to consequence simulation and resource optimisation to information flow enhancement on the network members. Artificial intelligence techniques and predictive analysis methods act as multipliers that accelerate the innovation and development of military theory [3]. In Section 4, we examine new directions in the development of decision support systems. We discuss the properties of network decision support models and briefly summarise the main differences between network and hierarchical approaches, highlighting the aspects that give rise to major differences.

## 2 Machine learning methods in CBRN protection

In this section, we will point out the relevance of our study, we will discuss the structures of CBRN defence, the general scheme of machine learning (ML) solutions, and existing good-practise applications.

### 2.1 Literature review of ML solutions in CBRN protection

We focus on the CBRN framework, especially the components supported by machine learning techniques, autonomous optimisation, and decision support solutions. Therefore, we do not analyse task-orientated tools, sensing technologies, and medical inventions. We extensively reviewed the literature available from Scopus following the PRISMA-P methodology (Preferred Reporting Items for Systematic Reviews and Meta-Analysis Protocols).

**Figure 1:**
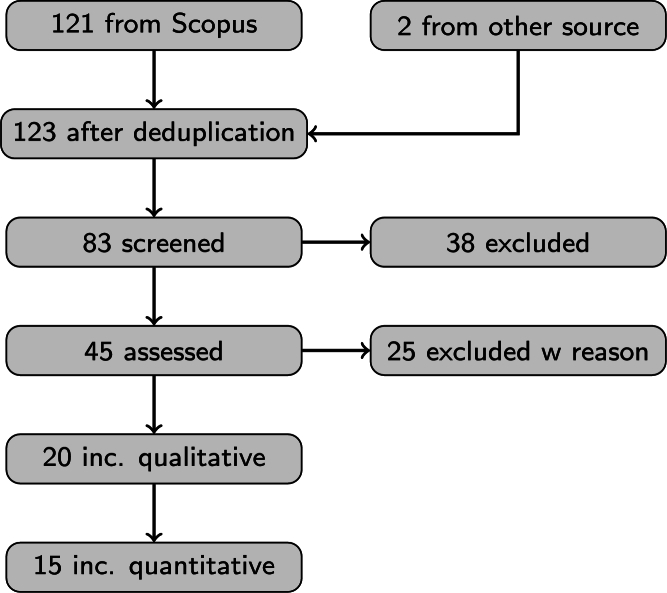
PRISMA processing flow of CBRN review articles. The diagram shows that out of 123 articles, there are only 15 identified as relevant review literature for a modern CBRN protection planner, but none of them discuss the useful machine learning techniques in detail.

By searching for the keyword of ((“CBRN” OR “CBRNE”) AND “REVIEW”), 121 articles were involved in the analysis from a Scopus search and 2 more from external findings. 83 articles were screened of them. Then 38 articles from the subject areas of Medicine, Biochemistry, Genetics and Molecular Biology, Nursing, Pharmacology, Toxicology and Pharmaceutics, Chemistry, Chemical Engineering, Physics and Astronomy were excluded. Further 25 articles were excluded because of weak connection to the CBRN subject and another 5 for qualitative reasons. Fig. 1 shows the change in the size of the evaluated literature in the PRISMA steps.

**Figure 2:**
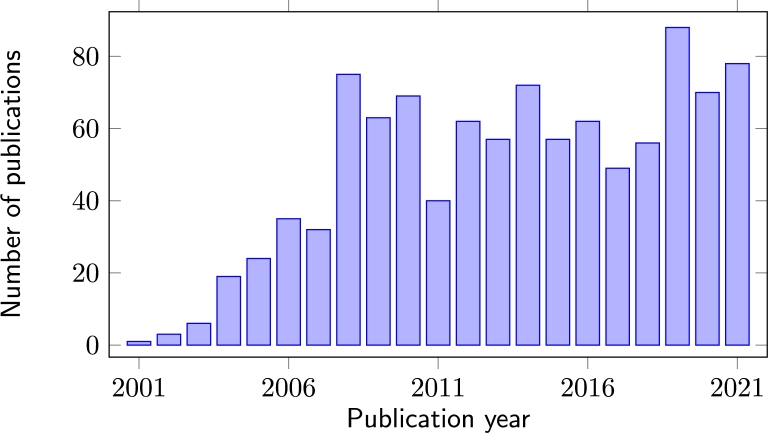
Distribution of CBRN(E) publications by years. The chart presents a continuous growth in the number of CBRN-related articles, and hence the increasing importance of the topic.

Fig. 2 presents the CBRN(E) related publications by years. It shows that CBRN problems and even more smart solutions are intensively researched topics, and the number of related publications follows a continuously rising trend.

We found a limited number of review articles that cover only a selected area of the CBRN processes: the human factors [4], the evacuation processes [5], the detection of biological warfare agents [6], communication issues [7], robotic technologies [8], threats decontamination technologies [9] and risk assessment [10]. None of these articles discuss in detail the role of machine learning and data-based decisions in the CBRN framework, which led us to summarise the importance of predictive analytical methods in a modern CBRN solution.

**Figure 3:**
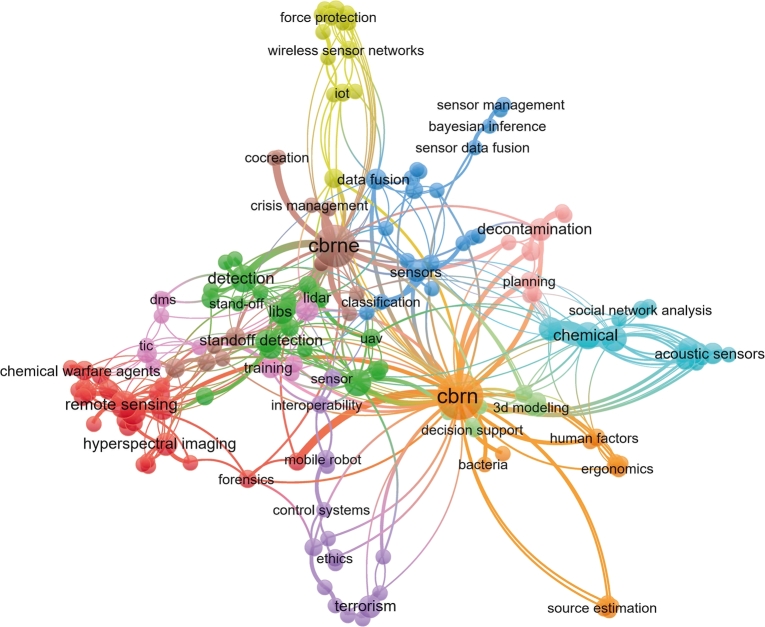
CBRN keyword co-occurrence analysis. The diagram shows the identified segments of publications by keyword co-occurrences. Practically, all segments have keywords with strong relations to machine learning and artificial intelligence techniques, which illustrate their power for efficiency improvements.

Fig. 3 shows the keyword occurrence map of CBRN(E) related publications without searching for review-type articles but applying the same subject area filter as in the PRISMA-P description. We can observe that predictive analysis techniques are widely used in almost all CBRN defence solutions processes. Practically all the segments contain integrated statistical modelling methods. Cluster 5 contains the foundational and general techniques: artificial intelligence [11], modelling [12], data fusion [13], classification [14]. Cluster 2 covers the sensing-related activities: remote detection [15], image processing [16], and robotics [17]. Cluster 3 includes the wireless sensor network [18], wireless communication [19] and IoT [20] technologies. Cluster 6 stands for virtual environment [21] and simulation [12]. The CBRNE sensing [22], detection [23], and standoff detection [24] belongs to Cluster 4. The orange cluster contains source estimation [25] and plume modelling [26]. Cluster 10 covers further sensing methods and processing procedures: social network analysis [27] and neural networks [28]. Cluster 1 supports operations with control systems [29], regression modelling [30], and risk management [31]. Finally, Cluster 12 includes the 3D modelling [32], information system [33] and decision support solutions [34]. Table 1 summarizes the dominant keywords of the identified clusters and the highest eigenvector centrality of the clusters, and hence the major directions of the CBRN researches and developments. Even there are significant ongoing developments in specific CBRN tasks, we can conclude that maintaining a modern CBRN defence system without data-based statistical modelling is impossible.

**Table 1:**
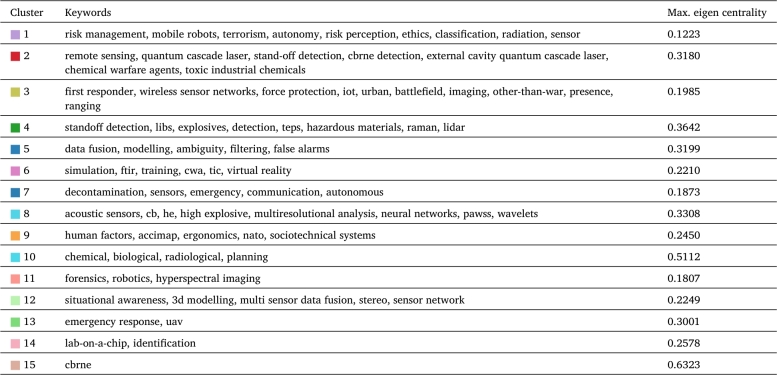
Dominant keywords by literature clusters.

The majority of the publications discuss only a particular part of the CBRN processes. There are focused reviews that summarise the key results of a selected area of CBRN protection. But general structuring of ML applications is not presented in CBRN protection and in the overall processes. This strengthened our motivation to prepare an end-to-end overview of an advanced CBRN protection process from sensor planning to decision support.

### 2.2 Functional structure of CBRN protection

CBRN protection is a highly diverse set of tasks and steps, but can be divided into five main functional areas [35].

1. *Detection*: detecting a CBRN incident or contamination, determining its extent, and monitoring its change over time. The main tasks of CBRN detection are detection, identification, and monitoring. Detection is the detection of a toxic substance in the air. It can be done chemically or physically, with human or mechanical intervention, using one or more sensors, mechanical or digital sensors, or a combination of these. The main detection methods are as follows:
  • Handheld portable sensors
  • Smart sensors
  • Unmanned vehicles
  • Ground/air/waterborne surveillance devices
  • Electronic data sources
  • Human sensing
  Modern military sensor networks are typically significantly larger and more complex than civilian sensor networks [36], but despite significant improvements in recent decades, these sensors are still costly. On the contrary, low-cost sensors have emerged for civilian use and have achieved remarkable results through mass deployment [37].
  In terms of sensors that take measurements, mobile sensors are gaining ground over static, fixed sensors. Three types of sensors can be distinguished:
  • Non-controlled sensors are mostly installed on moving vehicles [38].
  • In the case of centrally controlled sensors, the device can be directed to the area to be inspected, i.e., a dynamic solution is developed that is controlled according to environmental measurements [39].
  • Autonomously controlled sensors are used without central control, but with local control, learnt from the measurement results.
2. *Information management*: the collection, processing and transmission of CBRN intelligence data, including information transmission and exploitation activities. The IT revolution of the last decades has radically changed the tools that can be used. The previously unimaginable abundance of data and computing power offers the possibility of replacing the human resources involved in CBRN protection with an increasing number of operations carried out by machines with precision, reliability, and low processing time that far exceeds human performance. However, the transmission of data from sensors to processing systems has to meet some requirements:
  • Error-free: Measurement data must be sent with the accuracy of sensor measurement without any possibility of alteration.
  • Low latency: Data must be transmitted in the shortest possible time from the measurement time instant.
  • Energy efficiency: Unlike the low-latency criterion, continuous data communication can be highly energy intensive. Optimising the data transmission frequency is necessary to increase the uptime of sensors, which are typically battery-powered.
  • Security: By the system's nature, data integrity and non-repudiation are essential.
  • Operational reliability: The detection system can only fulfil its primary purpose if it is in continuous operation, so ensuring this is also important.
3. *Physical protection*: enhances survivability, but reduces reaction ability and capability. Physical protection consists of [35]
  • Individual protection: provided to individuals in a CBRN environment by protective clothing or personal equipment.
  • Collective protection: ensures a CBRN hazard-free environment to perform critical work or to obtain rest and relief in order to sustain combat operations.
  • Equipment and material protection: Covers mission-essential equipment and materials protection from damage and the need for subsequent decontamination.
4. *Hazard management*: needs to limit the impact of CBRN incidents. It can be supported by prehazard precautions, avoidance, spread control, and decontamination. Hazard management requires preliminary preparations, and hence it should be an integrated part of all planning processes.
5. *Medical countermeasures and assistance*: provision of adequate medical care for human items suffering from CBRN hazards. Commanders and authorised personnel should make decisions on medical advice about appropriate and timely protective activities. The types of countermeasures to mitigate the effects of CBRN hazards are:
  • Field hygiene measures during operations
  • Specific prophylaxis against assessed threat agents in advance of a possible attack
  • Post-event medical intervention
  • Post-exposure vaccination
  • Restriction of movement of possible direct and indirect victims of transmissible agents

The remainder of this article will focus on the efficient organisation of information processing and decision support processes. A prerequisite for this is understanding the CBRN detection methods, which are presented in more detail below.

### 2.3 Machine learning solution's development scheme

The main functional areas of CBRN protection determine the answer to what to do but do not specify how. In this section, we will summarise the general scheme of machine learning (ML) solutions, and present the key CBRN problems for which an ML method delivers a proven solution.

Today, new technologies and methods are emerging that lead to significant performance improvements and efficiency gains in many areas of industry and the economy. Artificial intelligence, machine learning, robots, smart devices, self-driving vehicles, drones, virtual reality solutions, nanotechnology, and synthetic organisms, among others, are transforming our lives so significantly that this period is referred to as the fourth industrial revolution. Exploiting all of these opportunities has also begun in the field of CBRN protection and is likely to become a more sustained process due to the significant untapped potential. Below, we review the general structure of a machine learning system and then several ‘good practise’ applications of new technologies in CBRN protection.

There are four interdependent phases in the use of machine learning systems [40]. The first phase includes source data generation operations. To properly exploit the potential of machine learning, the first requirement is to have the right amount, quality, scope, and frequency of data, which must be combined and then integrated.

The second phase of machine learning solutions consists of data storage, processing, and preparation steps. Since the input data requirements of different machine learning methods are different, the data structures and machine learning methods can mutually constrain each other. Supervised learning methods with limited autonomy can be applied to well-structured data. For image and sound data, reinforcement learning methods can be used, whose adaptive self-learning function can be adjusted to the situational experience. Natural language processing algorithms can be used for all types of sound recognition, sound interpretation, and sound orientation analysis. Neural networks support Big Data operations the best in processing large amounts of data. In addition, mechanical sensors can be integrated with IoT devices to accelerate data flow and, in many cases, provide remote control.

**Figure 4:**
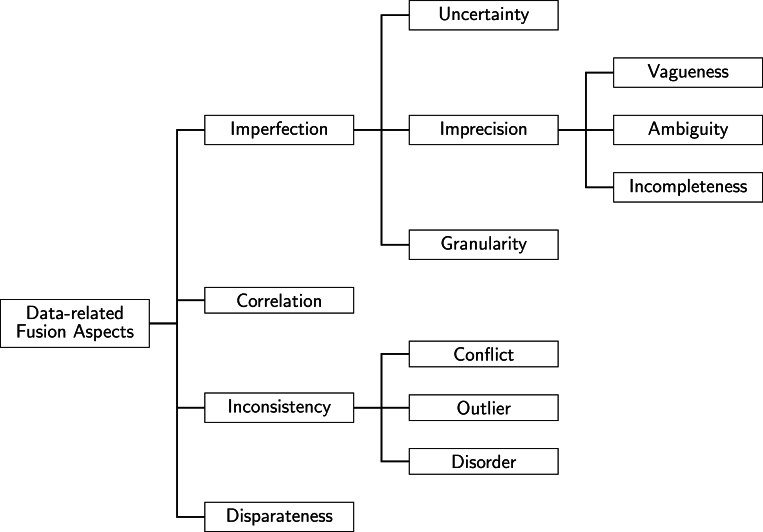
Taxonomy of data fusion methodologies The chart describes the types and sources of measurement errors. By taking these into account the CBRN system can be prepared for estimating and handling measurement errors during the data processing progress.

The third phase of machine learning systems includes data fusion and model-building tasks. According to the experts, the developed models can only be as accurate as the source data, and the importance of striving for the highest data quality cannot be overemphasized. Fig. 4 illustrates the types of data fusion error sources [40]. The four major error sources are measurement imperfection, correlation, data inconsistency, and disparateness. Measurement imperfection can be caused by uncertainty or the imprecision of the measurement instrument or its granularity. We can also distinguish the source of the measured data inconsistency, which can be derived from conflict, outlier, or disorder. Of these, the types of measurement imperfection are essentially unavoidable, which can be prepared for by considering the measurement accuracy ratio defined in the measurement instrument qualification. Furthermore, by designing the installation of the measuring devices and by selecting the measuring devices appropriately, it is possible to ensure that the measurement errors are within the desired tolerance range. To a large extent, the handling of correlation and imbalance errors is also a design issue, while the identification and elimination of inconsistencies can be addressed in the validation steps of the data processing procedures.

It is essential that the data processing process includes a robust adaptive programming framework that can correct for data diversity, imperfections, inaccuracies, and other sources of error.

The fourth phase of machine learning systems is information-sharing operations. In the context of CBRN defense, this includes not only the decisions made but also the derived and computed information generated in the upstream parts of the process. In recent years, new approaches have gained ground in this area, which is discussed in Section 3 and Section 4.

Machine learning methods, machine intelligence, and smart solutions are extremely diverse, but below we present some of the solutions that have been successfully applied in the context of CBRN protection and have high potential:

• *Smart devices (IoT), sensor networks*: traditionally, CBRN detection has been performed by specific target devices operated by human personnel. This allowed the detection of the types of contaminants under investigation even at low concentrations, but the deployment of personnel to the study area, the performance of measurements, and possible laboratory evaluations required significant lead times in a situation where every minute counts. In addition, the number of human units capable of such a detection task also limits the CBRN detection capabilities.
  An effective response to these challenges is provided by Internet of Things (IoT) sensors, sensor networks, and sensor clusters. The underlying idea is that faster response times are possible by reducing the CBRN detection time. This is achieved mainly through the development of Early Warning Systems (EWS), which are based on sensors and sensor networks with lower accuracy but more extensive coverage [41]. Furthermore, sensor clusters that combine different sensing technologies are gaining increasing attention [42]. In both cases, the aim is to enrich the measurement data, from which statistically significant conclusions can be drawn during processing, at a lower cost and with shorter detection times compared to human detection.
• *Sensor fusion*: modern CBRN protection solutions use several different types of sensors in parallel. These include long-range sensors such as radar, infrared, and electro-optical devices, short-range sensors such as Raman spectrometers, and point sensors such as ion mobility spectrometers (IMS) and chemical agent analysers. One of the critical issues in CBRN detection is how to integrate the different measurements to obtain the most accurate position assessment. Using sensor fusion techniques, a low parameter aggregation model has been developed that estimates the extent of the contamination cloud by combining the measurement results from different types of sensors [43]. Ensuring the integrity of the CBRN protection information flow does not eliminate possible measurement errors, but indicates its low confidence by low values of the reliability indicators. The method is easy to automate and allows real-time data processing, but requires prior parameterisation of the model. Human observations can be integrated into the model using a sample of point-based measures.
• *Modelling the dynamics of pollution clouds*: pollutants entering and leaving the atmosphere are usually not concentrated, but have a long-lasting, large-area impact, which makes it essential to use methods that provide the most accurate picture of the likely consequences of disasters as quickly as possible in decision-making processes [44]. To determine the atmospheric dispersion of pollution, various weather data are needed: wind direction, speed, variability, diurnal pattern, vertical profile; temperature stratification; relative humidity and precipitation; and atmospheric stability. These data are essential for determining the dynamics of pollution, which also shows that it is not sufficient to measure only the presence and extent of pollution in the sensing layer, but that additional sensing, such as weather sensing, is also required. Based on measured values and forecast models data, synoptic specialists produce a coded message containing ground meteorological information (CDM), but messages can also be produced from forecast fields without human intervention using algorithms [44].
  Several automated CBRN pollution modelling software is already in live use, typically with the following functionality: generation, transmission, reception, and processing of CBRN messages in a standardised format; data transmission according to different protocols; assessment of CBRN precipitation and nonpollution emissions, calculation of radiation dose and level, geospatial visualisation, exercise planning, built-in emergency response manual. During the processing, a variety of machine learning models optimised for the specific subtasks were used, the detailed description of which is beyond the scope of this paper. What is important to highlight is that near-real-time machine modelling of the dynamics and extent of contamination clouds mobilises computational and pre-learning capacities that are beyond the reach of human experts.
• *Simulation decision support*: when a CBRN event occurs, decisions have to be made in a highly complex situation, which is very demanding for the staff involved in the process because they have to react optimally to an uncertain, complex, and dynamically changing situation. This is compounded by the psychological burden of knowing that their decisions may directly affect human lives. In addition, CBRN events are rare events with a high impact but low probability of occurrence and consequently are difficult to typify and not really comparable with each other because they occur in different geographical locations and under different circumstances. All of these factors support the need for the most comprehensive information possible in CBRN protection decision processes to minimise the risk of erroneous decisions [45].
  To do this, the system uses the data and information available and generated during the previous steps of the process to classify the rules of the current operating procedure according to their relevance and perform probabilistic calculations on the consequences of the decisions. In doing so, it provides significant support to the relevant decision maker by identifying the appropriate operational steps and quantifying the probability of each scenario occurring.
• *Virtual twin environment*: CBRN protection training is usually conducted as physical exercise, which is essentially necessary and useful, but also time consuming and costly. Digital twins, which are virtual models of the plant, are increasingly being used to analyse and optimise the operation of industrial manufacturing plants. This allows complex analyses of changes on the shop floor to be carried out quickly and cost-effectively. Unfortunately, creating a full virtual replica of the CBRN-protected areas would be a task beyond reality, but virtual twin environments have been created for training exercises [46]. Virtual reality, mixed virtual reality, and personal computer solutions have been developed and evaluated with several participants who have previously performed physical exercises. The results suggest that virtual twin environments could play an important role in future CBRN protection training.

### 2.4 Modular CBRN protection framework

**Figure 5:**
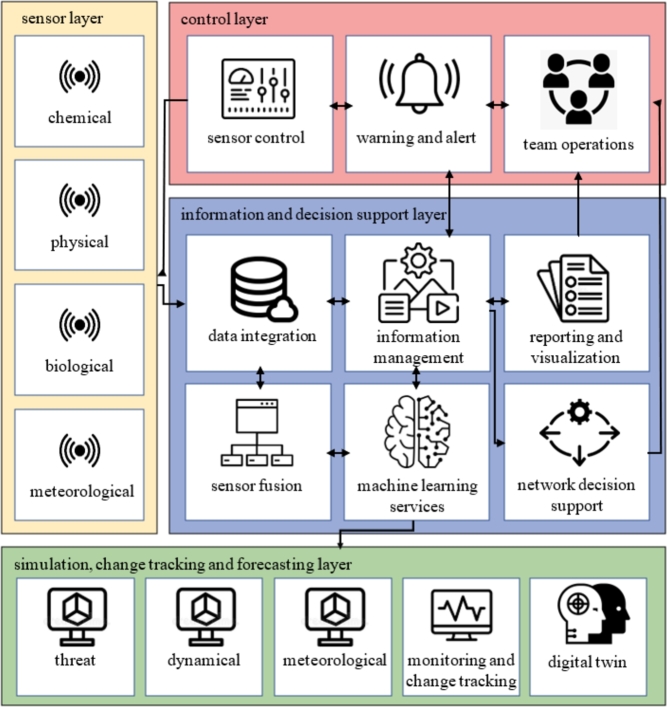
Demonstrative CBRN protection framework. The illustration covers a general modern CBRN system by defining fundamental tasks and organizing them into layers. Such a system can be built modularly, and hence results an ongoing development.

After getting to know the main functional areas of CBRN protection and ML solutions, we will summarise a modern CBRN framework. The most recent publications are integrated into our results. So we processed the Polish model [47], the Swedish model [48], a Norwegian approach [49], the findings of the pan-European EU-Sense project [50], and the NATO concept [51], but our framework differs from them in several aspects. We emphasised that the framework in Fig. 5 is a general demonstrative prototype model, which shows how complex is a CBRN protection system and how widely are ML methods integrated into it. We organised the elementary CBRN tasks into layers. The *sensor layer* covers physical and chemical detection operations, including chemical, biological, and nuclear detectors, and IoT tools, as well as meteorological sensors. *Information and decision support layer* contains data integration, sensor fusion, and information management steps. It provides machine learning services for a wide range of operations. It also involves network decision support mechanisms and reporting and visualisations functionalities. The *simulation, change tracking, and forecasting layer* stands for different simulation techniques of threat's extent and dynamics, meteorological tendencies, monitoring and change tracking tasks, and by learning the effect mechanism, dedicated digital twin environments. Finally, the *control layer* covers the sensor control solutions, the warning and alert systems, and team operation steps. The appropriate organisations could and should customise the framework to reach an optimal version for their own.

### 2.5 Formulating decision situation for automating artificial support

**Figure 6:**
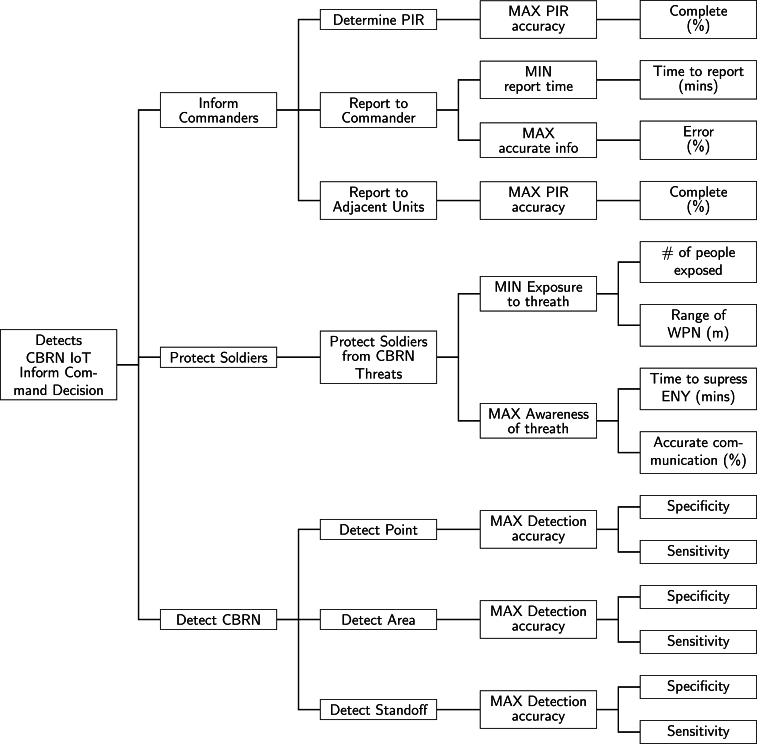
Functional structure of CBRN decision support system. The chart breaks down the CBRN protection decisions into measurable subgoals, which is essential to integrate machine learning solutions for improving and automating subtasks in a complex decision situation.

The central collection of measurement data enables the extraction of information and the execution of decision support operations, which is the most critical point of CBRN protection [52]. Fig. 6 describes the functional architecture of a CBRN protection decision support tool. There are three major types of CBRN Internet of Things (IoT):

• *CBRN detection tools*: the different IoTs enable to detect CBRN pollution source points, the contaminated area, and the endangered standoff. All the different tools have the objective of maximising detection accuracy, which can be measured due to specificity and sensitivity indicators.
• *Soldiers protection tools*: This kind of IoT supports minimising exposure to the threat by determining the number of people exposed and the range of WPN, and maximising awareness of the threat by estimating the time to suppress ENY and the probability of accurate communication.
• *Commanders information tools*: the primary objective of the third type of IoT tools is to inform commandants by determining the best-fitting priority intelligence requirement (PIR) by maximising the precision of the PIR, reporting to the commander by estimating the accuracy of the reporting time and the information, and finally reporting to adjacent units by maximising the precision of the PIR.

The key result of this approach is to formulate exact objective functions for the certainty of the relevance of priority detection requirements (PDR), notification times, degree of exposure to contamination, and detection certainty. This paved the way for the use of optimisation techniques already successfully applied in the industrial, economic and research fields. Such a solution enables speeding up the optimisation process and automating it, which leads to a highly sophisticated, near-real-time decision support tool.

### 2.6 Literature review and point of reference of machine learning applications in CBRN protection

Table 2 summarises the main components of the proposed CBRN framework of Fig. 5 and their ML methods applied by the sensing (SEN), information and decision support (INF), simulation, change tracking and forecasting (SIM) and control (CON). After describing the components' operation and goal, we collected the machine learning and machine intelligence technique by referencing the related literature. In the upcoming section we will discuss further methods to improve CBRN protection efficiency.

**Table 2:** Components and ML methods of CBRN sensor layer.

Layer	Component	ML methods
SEN	CBRN pollution detection layer: includes fixed and mobile sensors as well as human observers. It is heterogeneous in nature and type. Its purpose is to collect data on the area under investigation. Sensor technology uses encoder algorithms, communication optimisation methods, and network balance solutions.	network security [53], verification and authorisation, IoT [54]
INF	CBRN data integration layer: used to collect, consolidate and store raw data from the sensor.	decentralised military sensor networks [55]
INF	CBRN sensor fusion layer: integrates and aggregates data from different sensors and then determines the existence, nature, type, and extent of pollution. Its operation requires prior parameterisation and calibration. Additional data quality control and validation steps can be embedded in this layer.	error detection [56], built-in validation [57], on-field navigation [58], risk assessment [59]
INF	CBRN information management layer: the information dissemination and sharing layer covering the entire CBRN protection solution, from detection, through the definition of the relevant parts of the relevant regulations, to the derived and calculated information for decision support purposes, to the decisions taken, to the operations performed and in progress. It is designed as a highly automated platform and communication protocol with strict privilege management and built-in logging functionality.	network supervision [60], information flow management [61]
INF	CBRN defence machine learning services: a set of procedures and services that can be used in a bounded and loose manner to produce inferences and extensions derived from existing information through the models used in an objective and reproducible manner. As today's computational capabilities allow, they can perform the steps delegated to them in a fraction of the time required for human processing in solving problems of complexity far exceeding human capabilities. Given that the capabilities of machine learning can be used at many points in the process, they are included as a core service in the proposed architecture.	predictive models [13], approximation methods [62], feature selection methods [63], trained model utilisation, model validation, factor decomposition [64], multi-objective optimisation [65], computational modelling [66], real-time optimisation [67], genetic algorithm [68]
INF	CBRN visualisation and reporting service layer: a layer optimised to maximise the efficiency of information delivery, with a dynamic self-service information interface in addition to standard formats and policy-defined reports.	3d modelling [69]
SIM	CBRN simulation and prediction layer: predicts the expected extent and distribution of pollution using meteorological, geographic, and pollution dynamics data. In a more advanced version, it also produces assessments for different operational scenarios, from which the expected effects of the intervention and their consequences on human and mechanical resources are evaluated for decision-support purposes.	deep learning [70], time series [71], forecasting [72], predictive model developments [73], 3d modelling [74], distributed simulation [75]
SIM	CBRN monitoring and change tracking layer: performs dynamic determination of the temporal variation and spatial movement of pollution.	forecasting [76], information value measurement [77], significance testing [78]
CON	CBRN sensor control layer: controls mobile sensors to maximise detection efficiency and provide adequate coverage of related processes. It uses uncertainty estimations, interpolation approximation models, and optimisation methods with restricted resources.	resource optimisation [79], path planning [80], distributed control [81], predictive control [82]
CON	CBRN warning and alert layer: information mechanism covering the entire population involved in the CBRN protection process	impact analysis [4], event classification [1], anomaly detection [83], consistency analysis [84], early detection [85]

## 3 OODA approach in CBRN protection

An obvious idea is in military-related processes to turn to the observe–orient–decide–act (OODA) loop technique which is gaining ground because of its simplified and focused approach [86]. It provides a low-level decision mechanism in which both human and machine actors can perform effectively. Moreover, this technique originated from military and defence applications, leading to extensive references to applications [87] [88], therefore the question arises whether it could improve CBRN processes as well. In this section, we give an overview of the OODA approach and its applications in CBRN protection and highlight the analogies of CBRN, ML, and OODA structures.

The OODA model was not explained in detail by its author, Boyd, but introduced a new approach to the decision mechanisms. Since then, many applications have been described, and a common interpretation of the OODA loop has been born. An episode stands for the following steps:

1. *Observe*: Gather information directly or indirectly; collect internal and/or external data.
2. *Orient*: Data fusion from heterogeneous sources; data cleaning and consistency checks; taking advances from data processes.
3. *Decide*: Identify the options; evaluate them by focussing on the objectives.
4. *Act*: Perform decisions

**Figure 7:**
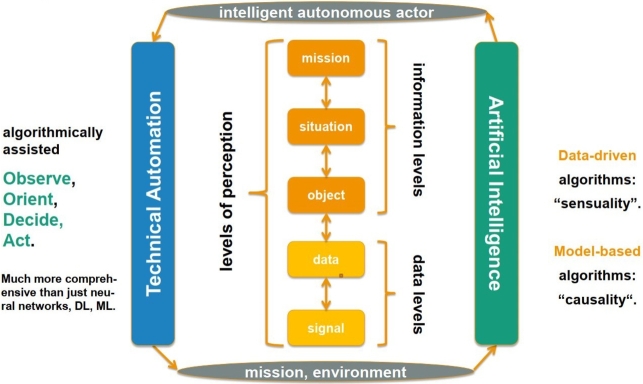
OODA application levels.

**Figure 8:**
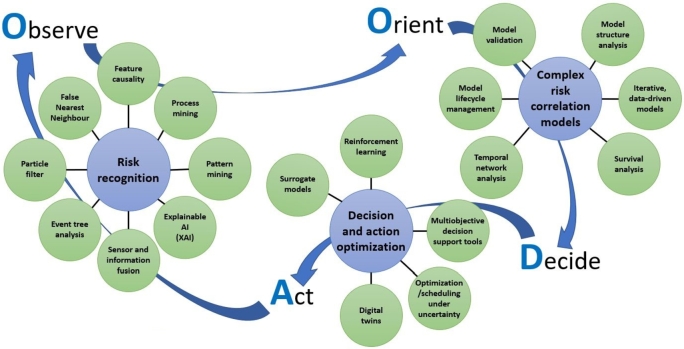
ML methods in OODA processes. The diagram shows the risk-based approach of OODA method and summarizes the ML methods assignments to the risk recognition stages.

Several processes of a CBRN solution can use the OODA loop. Fig. 7 shows the different perception levels on which the OODA approach is applied, whenever a decision should be made: directing the sensors remotely, cleaning the raw data and fusion it, managing objects, evaluating situations, or finally controlling the entire mission. From low-level decisions to high-level ones, the complexity increases. Nevertheless, decision processes can be supported by different ML techniques. Such an algorithmically assisted OODA approach is much more comprehensive than a pure ML solution. On the other hand, artificial intelligence agents can significantly extend the decision capability on all perception levels by delivering the most adequate suggestions within a reasonably short response time. The adequacy can be evaluated due to risk measurement. Bántay and Abonyi collected the methods most commonly used for CBRN-related OODA applications, which are presented in Fig. 8. They realised that OODA-based techniques can be effectively combined with risk-based approaches.

The observation steps are for risk recognition.

• *Sensor and Information Fusion*: To obtain a complete picture of the happenings in the environment, it is necessary to integrate the results of independent observation and to extract complex relations and general conclusions [13], [89].
• *Event tree analysis*: As the environment and the CBRN situations are quite complex, the relevant part of the state space can be discovered by extracting the implicit dependencies [90].
• *Particle filters*: Raw sensor data processing requires a large computational capacity and a longer response time. Particle filter-based methods help to reduce the number of relevant information sources to make reasonably intelligent decisions with a shorter response time even in extremely uncertain or incomplete detection situations [91].
• *False nearest neighbour*: One of the most challenging problems is to detect and classify partially covered objects in the data stream of sonar, lidar, or other sensing technology. Traditional image-based processing techniques cannot readily detect partially hidden objects. The false nearest neighbour algorithm on the transformed output times series of recurrence plot analysis can deliver an effective solution for these kinds of problems [92].
• *Feature causality*: Causality analysis is applied to select causal features of events / objects / goals-relevant variables to improve prediction performance and interpretability of adequate sensors [93].
• *Process mining*: Analyzing the tasks in an operation enables one to find best fitting sequences for the different situations, but furthermore, it is also applicable to improve human-machine communication by identifying the most relevant reference processes effectively [94],
• *Pattern mining*: The sensor networks produce a large amount of data streams that are dynamic, heterogeneous, and distributed. It is a complex task to process the data in real-time to extract high-value information from it like event identification, process monitoring, or faulty detection. Traditional data mining techniques cannot be used directly, leading to the application of behavioural pattern mining algorithms for sensor networks [95].
• *Explainable AI*: CBRN protection is a critical activity that cannot be fully delegated to machine intelligence. But for human interactions, the black box models do not deliver easily understandable relations discovered from the observations. Hence explainable models become more valuable [96].

The orientation steps require complex risk correlation models. The main ML approaches of behind are:

• *Model validation*: Statistical modelling extracts the knowledge of hidden patterns from the training data sets. The models can perform effectively only when the environment's rules are stable. But the environment can change more or less over time, necessitating regular validation of existing models to verify their relevancy and applicability.
• *Model structure analysis*: Most often, models are evaluated by the adequacy of their outputs. By fine-tuning the model parameters in a calibration process, the best-fitting model can be found in the continuously growing test data set, assuming that the model structure is optimal for the observed environment. New techniques come into the focus to find the best fitting model structure for the analyzed problem [97].
• *Iterative, data-driven models*: A sequence of model-based decisions provides the potential to interact with the predicted and the observed outcomes, and repeatedly refine the used models [98],
• *Survival analysis*: covers techniques to simulate multi-period decision series on the basis of single period outcomes and hence to find optimal strategies on longer time horizon [99].
• *Temporal network analysis*: CBRN threat evolves over time and, hence, can be modelled by dynamic methods. Temporal network analysis is a fundamental and flexible way of describing entities, their activities, and relations as an evolution of a complex system with graph-based techniques [100].
• *Ongoing model life-cycle management*: predictive and descriptive models is not a one-time task, but an ongoing process of training, validation, selection, deployment, monitoring, update, and termination [101]. When using a large set of models, this is a complex, important, and highly responsive activity.

The decision steps can be supported by

• *Digital twins*: to train the ML models in a simulated environment, and once the model performance reaches the target threshold, then it can be used in live processes.
• *Surrogate models*: Traditional optimisation models usually require a fairly long time, which results in some delay in reactions and operations. Therefore, rapid model development technologies, especially surrogate models, came to the fore. By having a reasonably good approximation function for the target variables, the simulations and what-if analyses preparation time can be significantly shortened. Although traditional optimisation methods can provide a better solution, surrogate models can obtain an approximate optimal solution in a much shorter time, and this can be very important for decision-makers in an emergency case such as a CBRN disaster [102].
• *Reinforcement learning techniques*: let agents learn episodically with a trial-and-error concept [103]. In some critical situations, trials cannot be carried out by your own actions, but with simulated or externally observed actions.
• *Multi-objective decision support tools*: enable the decision-makers to define multiple goals and to find a balanced solution that takes care of all of them parallel [104].
• *Optimization/scheduling under uncertain conditions*: Although there are extensively analysed problem types with efficient solvers, in practise the formulation is not strict enough or not deterministic. In such a case, optimisation and scheduling problems require special solutions under uncertain conditions. There are several approaches: robust optimisation methods consider the worst possible outcome and optimise decisions on that. Deterministic equivalent methods deliver an approximate formulation for a stochastic problem, which is easier to interpret and fits well with the problem. Recourse models enable corrections in current parameters whenever a better estimation can be concluded for an uncertain parameter. In CBRN prevention processes there is a wide range of uncertainty: identifying a biochemical component in the atmosphere, finding the shortest path after a disaster, or making the supply chain more robust under uncertain conditions [105].

Thus, decisions can be made according to the conclusions taking into account the results of the risk evaluation and simulation. The action steps must be performed, and its impact will affect the environment and the OODA cycle will repeat.

**Figure 9:**
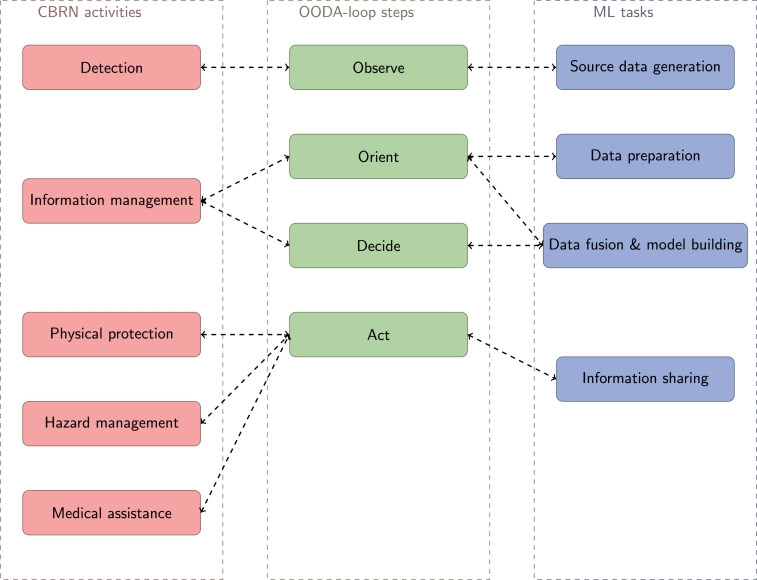
Relationships of CBRN activities, OODA-loop steps, and ML tasks. The chart highlights the similarities of CBRN protection structure, OODA-loop, and ML solution phases by connecting the corresponding steps.

Both the traditional CBRN protection structure, the OODA-loop, and ML solutions follow a common general decision scheme: First of all collect raw observation data, followed by transforming and structuring it. Then place it into a wider context to get conclusions for the optimal reaction. Finally, once a decision is made, it should be propagated to the affected participants. Fig. 9 shows the relationships between CBRN activities, OODA loop steps, and ML tasks.

## 4 Network decision support systems in CBRN protection

In this section, we will discuss the weaknesses of the different decision mechanisms, and present the potential of Network Decision Support System (NDSS) in CBRN processes.

The OODA approach was also criticized [106] because it often oversimplifies the decision situations, but numerous extensions were presented to extend and improve it to different decision problems. The major advances in using OODA methodology are to reduce decision time and be open to revising the assumptions and known pieces of evidence of the observed environment to conclude optimal decisions. The role of humans in decision processes cannot be completely eradicated because all the models have limitations and can be unprepared for rare situations [106]. Therefore, above a signification level, some human effort is required for making decisions. In such a complex solution as a CBRN defense system, the required human decision capacity is high. Hence a centralized decision-making organization/team cannot have sufficient capacity. This fact increases the importance of Network Decision Support Systems (NWDSS). Its basic properties are the following [107]:

1. *Flexible network with heterogeneous elements*. The concept is based on the structural design: the network nodes can be people, sensors, or software agents. Human nodes can form an entity individually or as a group. The latter typically refers to some organizational unit, such as a military unit, law enforcement agency, fire brigade, or various local and governmental institutional groups. Human nodes in a network usually contain decision-makers, although network intelligence can make certain low-level decisions through software agents, such as network management and control.
  The plasticity of the network is due to its constant change: people involved may switch off their phones, sensors may go offline, or go into silent mode. The interconnectivity of network nodes is also dynamic, so the exact structure of the network is in constant flux.
2. *Sensory abundance*. By definition, a network can contain human and non-human components as sensors. The miniaturization and explosion in the number of machine sensors are the main drivers for the emergence of networked decision support systems, particularly in the areas of disaster assistance, defense, and military operations.
3. *Simultaneous human-machine, machine-machine, and human-human interactions*. Unmanned sensors significantly increase the requirements for cooperative activities beyond human-human interactions. Traditional decision support systems have focused primarily on Human-Computer Interaction (HCI), but this has become unmanageable with the increase in the number of sensors. Human-sensor interaction is required when a device needs to be controlled or guided. Sensor-to-human communication is justified when the sensor detects the occurrence of a noteworthy event. Sensor-sensor communication can be used to control an unmanned vehicle. Finally, human-human interactions remain an essential channel for information flow.
4. *Open, generative and self-organising system*. The network decision support system can be freely connected to newer and newer vertices, as in many internet-based social platforms. The emerging system is generative in the sense that it operates according to simple rules, and its behaviour can be described by network theory. Solutions built in this way have typically been problem-solving-focused, but the novelty of the concept is in its usage for decision support. However, the civil-military information-sharing model can also be applied to the emerging network-based command and control domain by introducing a new risk management model built on top of an information filtering network [108].
5. *Knowledge networks and emerging knowledge processes*. In networked decision support systems, information can be generated through the collaboration of multiple human and non-human contributors, which can be explored to achieve different goals, such as identifying different types of incidents or monitoring a physical area. The quality of the resulting network knowledge is the key to the efficiency of the collaboration between the humans, sensors, and software agents involved. From a knowledge management perspective, any network can be interpreted as a knowledge network by measuring the collective information available at each node. The dynamics of information flows can evolve through the development of Emerging Knowledge Processes (EKPs), which can be improved by enhancing or improving the flows between knowledge hubs.
  Due to the plasticity of the network, knowledge flows are characterized by varying sets of contributors and their changing connectivity structure. The information knowledge profile of the contributors is not known in advance and emerges during operation as information flows become more stable. The critical knowledge flow in networked decision support systems is expert back-propagation, where decision-makers can access either human or machine knowledge bases.
6. *Agile, collaborative decision making on the edges*. Networked decision support systems, particularly for emergency response or tactical operations on the battlefield, can support decentralized decision-making processes. These situations are often chaotic, lacking prior scenarios, are full of high-risk cases and strong time constraints, and do not allow for classical hierarchical decision processes. According to this, the organization of the actors involved in the decision-making processes is typically flattened, and decision-making is not centralized but takes place agilely at the edges.
7. *Computer modelling and experimentation*. Generative networks, such as decision support networks, are often analyzed using computer models. A simulation of the network in a virtual environment can deliver results to fine-tune network rules to improve more efficient knowledge flows.

**Figure 10:**
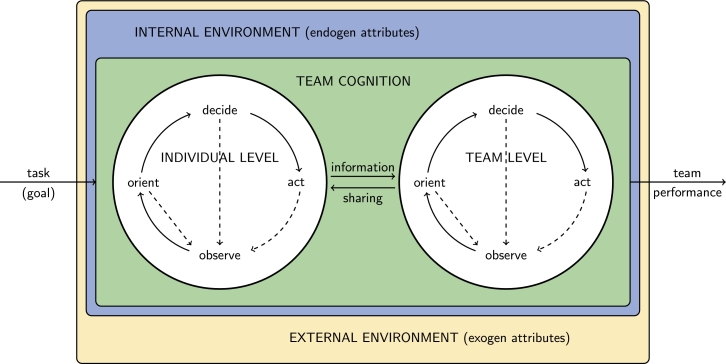
Team perception model. The diagram presents the concept of varying decision levels by using two-way information sharing. The model supports distributing the decision-making opportunity to a lower level under the team's control.

Inadequately controlled decision-making can easily lead to missteps [109]. To develop a well-functioning information-sharing protocol, it is worth considering its role. Today, it is a common phenomenon to use teams or organizational units of different sizes to achieve organizational goals. Teams consist of interdependent members who coordinate their work through a variety of interaction processes. These interaction processes are key to carrying out situational awareness and assessment even in dynamically changing environments. Accurate, timely, appropriately shared information is vital to the completion of team tasks, especially for task forces that are conducting complex or time-sensitive operations. The perception process model for teams is shown in Fig. 10 (Mullins, 2019). The strength of this approach is providing the opportunity to make certain decisions at a lower level, controlled by the team, using a two-way information-sharing platform. On the other hand, the team perception model provides a network-distributed decision mechanism, which is more robust and stress-tolerant than the centralized, scenario-based decision model.

From another aspect, CBRN protection processes are based on the situation evaluation and optimal responses to them. In general, there are a preliminary identified set of reference situations. It is a quite complex task to recognize the best-fitting situation and act according to that. The major difficulty behind this is that most of the current methods assess the situations at the moment and not as a dynamic system. Formally the situations are a set of physical entities, objects, and events, and their relations and the tendencies of their state change. The participant can get spot observations mainly about the former, but not about the latter, which can be discovered by AI and ML methods. Furthermore, the reference situations set requires ongoing revision and refinement.

**Figure 11:**
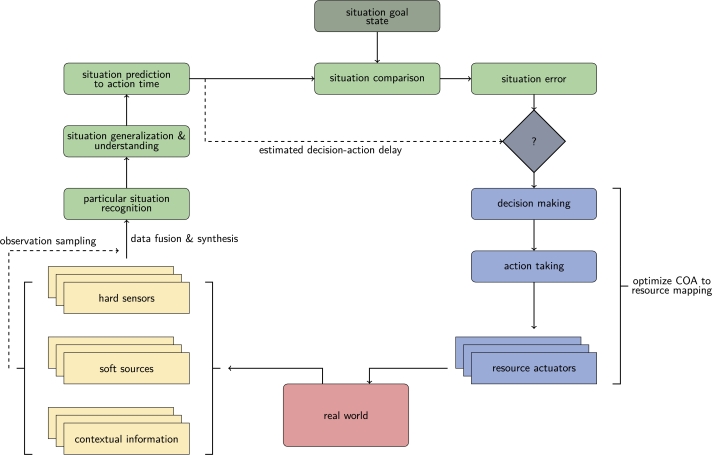
Cognitive situation control. The chart represents a process flow of getting observation samples from sensors and further information sources, recognizing the current situation, identifying the most similar known situation, and finding the optimal actions to reach the goal state in a continuous repeating cycle.

By going into the details, estimating situational dynamics needs situation recognition, prediction, evaluation, and refinement to build up an action-taking architecture. Fig. 11 shows the varied processes for all of these functionalities, which are integrated into a closed-loop “situation control” framework [110]. The major components of such a process are the followings:

• *Data collection*: hard information is sourced from sensors, soft information can be retrieved from external sources, and contextual information is extracted from processed data sources.
• *Data fusion and preparation*: information from all sources needs to be integrated, cleaned, and pre-processed. For faster processing, it is suggested to get a representative sample, which can be used for particular situation recognition.
• *Situation generalization*: the particular situation should be used for mapping to a preliminary defined generalized situation state.
• *Situation evaluation*: predicting the situation for action time horizon on the basis of operation tempo will provide an expectation for it. Then this can be compared to the situation goal state to get the situation “error”.
• *Decision and action taking*: to eliminate the gap between the situation expected and goal states the optimal decision can be made, and according to it, the necessary actions can be taken.
• *Effects*: the actions will cause some effects in the real-world environment, which needs to be observed, and the circle repeats again.

## 5 Summary and conclusions

There have been recent technological and operational breakthroughs in several areas that can effectively help improve CBRN protection. We gave an overview of the methods and the applications where these are already used and highlighted further potential. We structured the applications by layers and concluded it is important to properly design the information sharing and decision support layers of a hybrid (human-machine) detection network. To achieve this, it is proposed unidirectional information channels, incorporating validation and control mechanisms, supporting the smoothness of information flows, and integrating machine sensors and artificial computer agents. Over the layers, we collected the applicable machine learning methods and assigned them to the layer's applications. We showed that OODA methodology revolutionized the military decision-making processes, and a large proportion can be automated, but human control and responsibility are still necessary. For this, we presented the characteristics of network decision support systems. We highlighted a cognitive situation control loop, which points to a structure beyond static operational scenarios to respond effectively to unexpected situations, to make optimal decisions based on collective knowledge. We found that further developments have large potential in which machine intelligence will have an even more important role.

Although ML methods can improve CBRN protection capabilities significantly, the data collection, training, and tuning processes take a long time, and their integration into decision strategy requires hands-on experience of its reliability. Therefore, we suggest managing a continuous development system for improving systematically CBRN protection solutions by integrating effective ML applications into the concept.

## CRediT authorship contribution statement

**Tamás Kegyes:** Conceptualization, Formal analysis, Investigation, Visualization, Writing – original draft, Writing – review & editing. **Zoltán Süle:** Conceptualization, Methodology, Validation. **János Abonyi:** Conceptualization, Methodology, Supervision.

## Declaration of Competing Interest

The authors declare that they have no known competing financial interests or personal relationships that could have appeared to influence the work reported in this paper.
